# Common bean under different water availability reveals classifiable stimuli-specific signatures in plant electrome

**DOI:** 10.1080/15592324.2024.2333144

**Published:** 2024-03-28

**Authors:** Gabriel R. A. de Toledo, Gabriela N. Reissig, Luiz G. S. Senko, Danillo R. Pereira, Arlan F. da Silva, Gustavo M. Souza

**Affiliations:** a Laboratory of Plant Cognition and Electrophysiology, Department of Botany, Institute of Biology, Federal University of Pelotas, Pelotas, Brazil; b São Paulo State University, Presidente Prudente, Brazil; c Department of Physics, Federal University of Pelotas, Pelotas, Brazil

**Keywords:** Complexity, machine learning, osmotic stress, plant electrical activity, salinity, small vector machine

## Abstract

Plant electrophysiology has unveiled the involvement of electrical signals in the physiology and behavior of plants. Spontaneously generated bioelectric activity can be altered in response to changes in environmental conditions, suggesting that a plant’s electrome may possess a distinct signature associated with various stimuli. Analyzing electrical signals, particularly the electrome, in conjunction with Machine Learning (ML) techniques has emerged as a promising approach to classify characteristic electrical signals corresponding to each stimulus. This study aimed to characterize the electrome of common bean (*Phaseolus vulgaris* L.) cv. BRS-Expedito, subjected to different water availabilities, seeking patterns linked to these stimuli. For this purpose, bean plants in the vegetative stage were subjected to the following treatments: (I) distilled water; (II) half-strength Hoagland’s nutrient solution; (III) −2 MPa PEG solution; and (IV) −2 MPa NaCl solution. Electrical signals were recorded within a Faraday’s cage using the MP36 electronic system for data acquisition. Concurrently, plant water status was assessed by monitoring leaf turgor variation. Leaf temperature was additionally measured. Various analyses were conducted on the electrical time series data, including arithmetic average of voltage variation, skewness, kurtosis, Probability Density Function (PDF), autocorrelation, Power Spectral Density (PSD), Approximate Entropy (ApEn), Fast Fourier Transform (FFT), and Multiscale Approximate Entropy (ApEn(s)). Statistical analyses were performed on leaf temperature, voltage variation, skewness, kurtosis, PDF µ exponent, autocorrelation, PSD β exponent, and approximate entropy data. Machine Learning analyses were applied to identify classifiable patterns in the electrical time series. Characterization of the electrome of BRS-Expedito beans revealed stimulus-dependent profiles, even when alterations in water availability stimuli were similar in terms of quality and intensity. Additionally, it was observed that the bean electrome exhibits high levels of complexity, which are altered by different stimuli, with more intense and aversive stimuli leading to drastic reductions in complexity levels. Notably, one of the significant findings was the 100% accuracy of Small Vector Machine in detecting salt stress using electrome data. Furthermore, the study highlighted alterations in the plant electrome under low water potential before observable leaf turgor changes. This work demonstrates the potential use of the electrome as a physiological indicator of the water status in bean plants.

## 1. Introduction

Plants exhibit intense electrical activity, which plays a role in various physiological processes, beyond facilitating rapid movements in ‘sensitive’ and ‘carnivorous’ plants.^1–4^ Several electrical signals have been identified and well characterized.^5^ Among these, action potentials (APs), variation potentials (VPs), system potentials (SPs), and local electrical potentials (LEPs) are particularly notable.^6^ These electrical signals are extensively studied due to their signaling properties, enabling the rapid transmission of specific messages over short/long distances regarding internal/external changes, complementing plant chemical and hydraulic signaling.^7–10^

Thus, electrical signals, alongside other internal signaling mechanisms, are responsible for coordinating the physiology, metabolism, and behavior of plants, operating from individual cells to the entire plant organism.^11–14^ Plant electrical signals play pivotal roles in regulating various physiological processes such as photosynthesis,^15–19^ respiration,^20,21^ growth,^22^ stomatal aperture,^23^ and expediting defense responses against biotic and abiotic stressors,^8^ triggering cascades of gene expression alterations even in distant sites from the initial stimulation site.^24,25^

There are additional electrical activities present in plant life that may not necessarily be classified as signals, such as electron transport chains within chloroplasts and mitochondria, or the exchange of charges during nutrient uptake processes, electrical currents in growing pollen tubes, roots, and root hairs, as well as the photoelectric response of green leaves, among others.^26–31^ However, it is crucial to note that the majority of cellular electrical activity is facilitated by ion flux through membrane channels and pumps.^5,29,32^

The entirety of a plant’s electrical activity throughout its lifetime (or within a defined period across various tissues) can be referred to as the “plant electrome,” encompassing both electrical signals and other electrical events occurring within plant tissues.^30,33^ This terminology was adopted by our research group following insightful suggestions made by de Loof,^32^ who proposed this term to simplify the designation for the total electrical activity of any biological compartment, ranging from cells to entire organisms, over time, analogous to terms such as genome, proteome, transcriptome, and others.

The initial studies on plant electrome conducted by our research group involved soybeans subjected to various stimuli, such as low light, low temperature, and low osmotic potential, leading to intriguing findings.^30^ One significant discovery was the observation of a scale-free distribution of voltage variations and the presence of colored noises in the power spectral density (PSDs), indicating that the plant electrome may exhibit self-organized criticality properties under specific conditions, notably in instances of low light and low osmotic potential. Such characteristics are commonly associated with exceedingly complex dynamic systems, implying organizational aspects and functional optimization of information distribution and processing.^33,34^

The primary objective of our current research was to perform a more comprehensive characterization of the electrome using a different plant species compared to previous studies, seeking to investigate and compare similarities or differences with the soybean electrome.^33,34^ Hence, we selected a related plant species, the common black bean (*Phaseolus vulgaris*, L.), and applied distinct methods to stimulate variations in water availability, including the use of distilled water, nutrient solution, and the imposition of osmotic stress via water deficit and salinity. This approach aimed to thoroughly explore the plant electrome’s response to diverse stimuli.

Our primary hypothesis is centered on the notion that the electrome signatures of common beans are contingent upon the stimuli received, reflecting the environmental water status perceived by the roots. Specifically, it is anticipated that the electrome of common beans exhibits distinct characteristics associated with different types of water availability stimuli, discernible through various analyses, including automatic classification techniques. We postulate that the most intense and detrimental stimuli would induce the most pronounced alterations in the electrome. Considering that healthy physiological states are typically associated with higher complexity levels in biological dynamics, we also hypothesize that the electrome of plants subjected to the most harmful stimuli would display lower complexity levels. Moreover, if the electrome is indeed stimulus-specific, we aim to employ automatic classification algorithms to differentiate between stimuli based on electrome analysis.

## 2. Material and methods

### 2.1. Plant material and experimental conditions

Seeds of common bean (*Phaseolus vulgaris* L.) cv. BRS-Expedito, registered in the National Service of Cultivar Protection (number 00688), were used. It is an important cultivar for the economy and food security of southern region of Brazil, in addition to being well adapted to our experimental growth conditions.^34,35^

The seeds were sowed in Gerbox® boxes on Germitest® paper moistened with distilled water and kept under artificial lighting through a customized light system with LEDs adjusted to provide 500 µmol m^−2^ s^−1^ of photosynthetic photon flux density (PPFD). The photoperiod was 14/10 h and temperature 28°C ±2, controlled by an air-conditioned set. After three days, the germinated seeds that presented roots of 1 cm were transferred to 120 mL polystyrene cups filled with 150 g of washed and sterilized sand, pre-irrigated with half strength nutrient solution.^36^ The seedlings were irrigated once a day with this same nutrient solution, until seeping from the cup bottom, maintaining the substrate near the maximal water retention capacity. This proceeding avoided both starvation and stress caused by salinity or water lack/excess. The seedlings were grown under such conditions for 10 days. Then, three of them were transferred to a Faraday cage where the experiments were carried out.

**Figure 1.**
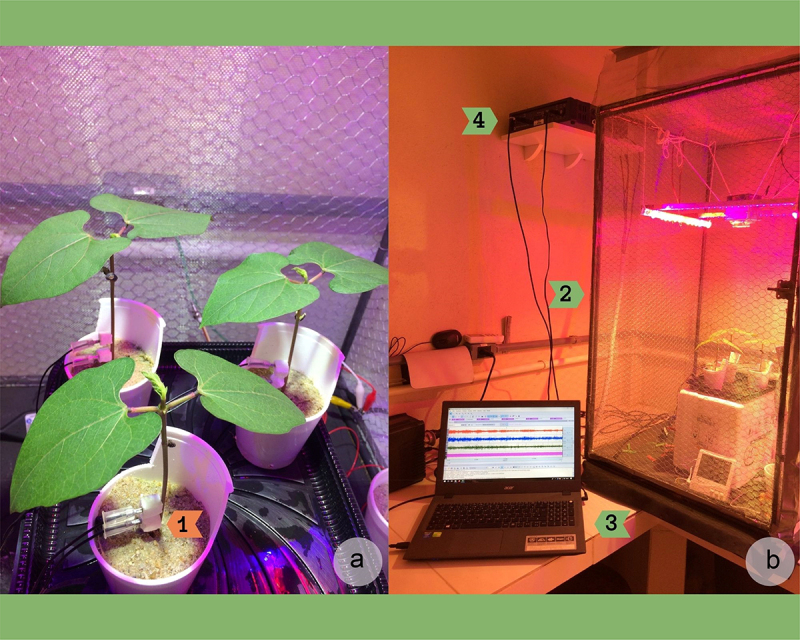
Experimental setup. (A) An experimental session with 3 healthy plants and a pair of needle electrodes inserted in bean stem’s basis (1), inside the Faraday’s cage. (B) External view from the Faraday’s cage and the experiment (2). Over the laboratory bench is the notebook with the electrophytograms (3) obtained in the experimental session. Connected to them is the electronic system MP36, used for electrical signal recording (4).

The Faraday’s cage was in the same experimental room where common bean plants were grown (Figure 1). Thus, the previous environmental conditions were kept during experimental sessions. The air temperature and relative humidity were monitored inside the Faraday’s cage in experiments I, II and III, using Yara© ZIM-sensors (ZIM-probe, YARA – ZIM Plant Technology GmbH, Hennigsdorf, Germany) situated close to the leaves. The needle electrodes (model EL-452) were inserted one day before the experimental sessions, to acclimate stem’s tissues from needle’s insertion.^5^ One pair of electrodes was transversally inserted, 1 cm apart from each other, in the stem’s base of each plant, crossing the whole stem diameter to monitor all electrical activity of all the cells/tissues situated between electrodes. The parts of the needles that were not inside the plant were covered by an insulating piece of polystyrene block (Figure 1a). This procedure allows to sample the bioelectric activity of a large number of cells (rather like working as an “internal” surface electrode), which is representative of the plant electrome, especially due to this region of the stem probably being a route to systemic and long-distance electrical signals originated in roots/shoot.^8^ In addition to the pair of electrodes used in each plant, a third electrode (ground) of each channel was connected to the Faraday’s cage. The cage was also grounded.

Three plants were used simultaneously to monitor the electrome dynamics in each experimental session (Figure 1a). A fourth pair of electrodes was used to monitoring the sum of intrinsic and extrinsic noise in the cage. This pair of reference electrodes (RE) was inserted in a common’s bean in the same way described for the other 3 pair of electrodes, but the segment of the stem was cut away and let it die and dry with the pair of electrodes inserted. This segment of the stem was used in all experimental session as reference electrode for intrinsic/extrinsic noise monitoring. All the experimental sessions consisted of three plants and one RE due to the number of channels (4) of the device employed to acquire the bioelectric activity (MP36, Biopac systems, Goleta, Ca, USA).

In total, four experiments designed to offer alternative ways of water availability stimulation were carried out, offering distinct but similar osmotic stimuli to characterize its effects on plant electrome. In this sense, it was opted to stimulate plants with two non-damaging stimuli with high water potential, (I) distilled water and (II) half-strength Hoagland’s nutrient solution. Oppositely, the plants were subjected to (III) PEG and (IV) NaCl solutions both with −2 MPa of water potential to provide two different harmful stress stimuli with the same intensity. Among the four stimuli, water provides the highest water potential to the plants. The nutrient solution also offers a high-water potential to the plants but lower when compared to distilled water, and higher than PEG and NaCl stimuli. PEG stimulus offers low water potential, simulating concomitantly quick drought and osmotic stress to plants while salinity also can induce salt toxicity beyond osmotic stress. The details of the four experiments are described in Table 1.

**Table 1.** Treatments with alternative water availability stimulation in common bean plants (*Phaseolus vulgaris* L.).

Treatments (stimuli)	Repetitions	Protocol	Measures
Water (I)	30 plants/10 sessions	20 mL of distilled water was carefully applied in each polystyrene cup, over the substrate, using a graduated pipette, without touching anything. The water was at the same room temperature to avoid additional stimuli).	Electrophytograms, leaf turgor, and environmental conditions were recorded simultaneously along 4 h in each experimental session. The leaf temperature was measured immediately before stimulation and 2 h after it.
Nutrient solution (II)	21 plants/7 sessions	20 mL of half-strength nutrient solution was applied in each polystyrene cup, over the substrate, using a graduated pipette, without touching anything. The nutrient solution was at the same room temperature to avoid additional stimuli.	Electrophytograms, leaf turgor, and environmental conditions were recorded simultaneously along 4 hours in each experimental session. The leaf temperature was measured immediately before stimulation and 2 h after it.
PEG (III)	54 plants/18 sessions	20 mL of polyethileneglycol-6000 (PEG) solution (with −2 MPa water potential) was carefully applied in each polystyrene cup, over the substrate, using a graduated pipette, without touching anything. The PEG solution was at the same room temperature to avoid additional stimuli	Electrophytograms, leaf turgor, and environmental conditions were recorded simultaneously along 4 h in each experimental session. The leaf temperature was measured immediately before stimulation and 2 h after it.
NaCl (IV)	39 plants/13 sessions	20 mL of NaCl solution (−2 MPa water potential, containing 28 g L^−1^ of NaCl) was carefully applied in each polystyrene cup, over the substrate, using a graduated pipette, without touching anything. The NaCl solution was at the same room temperature to avoid additional stimuli.	Electrophytograms were recorded along 4 h in each experimental session. The leaf temperature was measured immediately before stimulation and 2 h after it.

### 2.2. Electrophytography data acquisition

To monitor the plant electrome, the electrophytography (EPG) technique was employed.^34,37,38^ Electrical signals were captured within a Faraday cage using the MP36 electronic data acquisition system (Biopac Systems, Goleta, CA, USA), comprising four channels with high input impedance (10 GΩ). The MP36 was configured following electrocardiography (ECG-AHA; 0.5–100 Hz) standards, utilizing a sampling rate of 62.5 Hz. This setup included two filters: a high-pass filter of 0.5 Hz and a low-pass filter of 100 Hz. Additionally, a 60 Hz band-stop filter was incorporated to mitigate power line interferences.

The bioelectrical runs were analyzed as voltage variation (mV) time series ΔV = {ΔV_1_, ΔV_2_, … , ΔV_N_} in which ΔV_i_ is the difference of potential between the inserted electrodes, scored in each $\frac{1}{f_{s}}$ time interval, and N is the total length of the series. Considering the sampling rate and the duration of data acquisition (2 hours), time series (TS) containing 450,000 observations (62.5 × 60 seconds x 60 minutes x 2 hours = 450,000 points) were acquired. In total, 288 TS (60 in experiment I; 42 in II; 108 in III; and 78 in experiment IV) were collected and utilized for characterizing and analyzing the plants’ electrome (electrophytograms). Additionally, 96 TS (20 in I; 14 in II; 36 in III; and 26 in experiment IV) were used to analyze the reference electrode signals (data not shown) across the four experiments. Thus, the electrophysiological data exclusively analyzed in the four experiments presented in this paper amounted to 172,800,000 data points.

### 2.3. Plant water status and environmental conditions

The plant water status was monitored by leaf turgor variation using Yara© ZIM-probes (ZIM-probe, YARA – ZIM Plant Technology GmbH, Hennigsdorf, Germany) and by leaf temperature measurements using thermography (FLIR E-5, FLIR Systems, Wilsonville, Oregon, USA). One leaf turgor probe was installed in a simple leaf of each plant one day before experimental sessions start, following the device usage recommendation guide.^39^ The data acquisition frequency of ZIM-probes was set to 10 s in Yara® water sensor user’s page. Regarding leaf temperature, the thermographic pictures were taken 50 cm distant from the leaves’ surface. The temperature measurements were conducted using FLIR Tools software (Teledyne FLIR©, registered), calculating the average temperature along a line crossing the leaf longitudinally. However, in experiment IV, it was not feasible to monitor the environmental conditions and the plant water status due to the lengthy installation process of the probes. This time-consuming installation coincided with the nearing deadline for the Ph.D., making it impractical to complete the necessary measurements within the allotted time frame.

The air temperature and air relative humidity were monitored by Yara© ZIM-sensors (ZIM-probe, YARA – ZIM Plant Technology GmbH, Hennigsdorf, Germany), according to the procedures described in the section 2.1 (Table 1). The data acquisition frequency of ZIM-sensors was set to 10 s in Yara® water sensor user’s page.

### 2.4. Data analysis

#### 2.4.1. Visual analysis and characterization of electrophysiological time series

The electrophytograms were subjected to visual analyses to detect patterns associated with stimuli. Despite being imprecise and subjective, it allows the first analysis of time series and the observation of differences among treatments.^34^

Then, all electrophytograms were subjected to standard statistical analysis by calculation of: (1) arithmetic average of voltage variation values, (2) skewness, (3) kurtosis, (4) Probability Density Function (PDF’s α exponent measurement), (5) autocorrelation, (6) Power Spectral Density (PSD’s β exponent measurement), (7) Approximate Entropy (ApEn), (8) Fast Fourier Transform (FFT), (9) Multiscale Approximate Entropy (MAE). All these analyses were performed following the methods used in previous works with plant electrome.^33,34,40,41^

#### 2.4.2. Comparison of means and coefficient of variation

Leaf temperature, voltage variation, skewness, kurtosis, PDF µ exponent, autocorrelation, PSD β exponent, and approximate entropy data were subjected to statistical analysis. The mean of the values obtained before and after each stimulus (dependent variables) was compared using the paired t-test (*p* ≤ .05). In cases where the data did not exhibit a normal distribution, the Wilcoxon Signed-Rank test (*p* ≤ .05) was employed for analysis (Sigmaplot 12.0, Systat Software Inc., USA). Additionally, the coefficient of variation (CV %) was calculated for further assessment.

#### 2.4.3. Machine learning classification methods on electrome time series

Machine learning algorithms were used to realize the automatic classification of plant electromes under different water availability of the four experiments. The Algorithms used were Bayes classifier;^42^ Support Vector Machine (SVM);^43^ K-Nearest Neighbors (KNN);^40,42^ Artificial Neural Network (ANN);^40,42,44^ Decision Trees (DT);^42^ and Random Forest (RF)^45^).

Alternative combinations of these algorithms were used to do the classification of the electromes recorded before and after each stimulation and among the four different experiments after stimulation. Thus, the automatic classification was done to find possible differences among the electromes recorded before and after stimulation in each experiment and among the four experiments.

The Interval Arithmetic (IA) was used associated with the automatic classification to decrease the feature space, enabling better classification results. For such, each time series was divided into windows, from which the minimum, maximum and average values were extracted. Consequently, each interval (I) is represented by a triplet, where I = [*min*, *med*, *max*]. Thus, each original time series of 450,000 points (S) was divided into 15 intervals with 30,000 points each, where each one was represented by the trio formed by the average, the minimum, and the maximum, as follows: S’ = {[*min*_*1*_, *med*_*1*_, *max*_*1*_], [*min*_*2*_, *med*_*2*_, *max*_*2*_], …, [*min*_*15*_, *med*_*15*_, *max*_*15*_]}. Thus, the original time series has its feature space size reduced from 450,000 to 15 (450,000/30,000 = 15). The IA was applied before the classification process.^30,40,41^

The feature space reduction contributes to decreasing the computational processing demand in addition to decreasing the electrome heterogeneity, despite the information loss inevitably associated with this process. Other window sizes were tested (1000, 5000, 15000), however the division of the original TS into 15 intervals of 30,000 points each provided the best results. After subjecting the original TS to interval arithmetic, the data was subjected classification algorithms. This procedure was performed similarly to that described by Pereira et al..^40^ To perform the automatic classification of the bean electrome under different stimuli, a data set for training the algorithms was provided. Thus, each time series already submitted to AI was cut into training sets varying between 10% and 90% of the data to feed the classifiers. The process of reigning each algorithm with each data set was repeated 20 times to perform the Wilcoxon statistical test, like that performed by Pereira et al.^40^

As the intention is to detect patterns in the electrical activity of plants associated with stimuli, the algorithms training was performed using the data “before” and “after” the stimulus in experiments I to IV. In experiments I, II, and III, the performances of four classifiers (K-NN, DT, RF, and Bayes) were tested, while in experiment IV three algorithms were tested (K-NN, ANN, and SVM). Finally, the best classifier with the best performance using the smallest possible dataset for training was chosen to perform the validation through the confusion matrices. To generate the confusion matrix, data from different situations (such as “before” and “after”) are inputted mixed to the classifier. This allows verifying whether the classifier can in fact distinguish the patterns associated with each situation. Nevertheless, a confusion matrix was generated using the “after” data from the four stimuli to evaluate the classifier’s capability to discern patterns among them. This test aimed to determine whether the classifier could differentiate the various patterns associated with different stimuli, even when the data were presented to the classifier concurrently, mirroring the methodology employed by Pereira et al.^40^

## 3. Results

**Figure 2.**
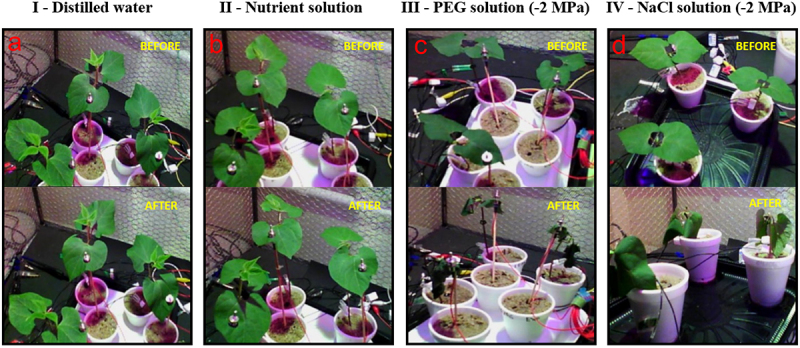
Examples depicting experimental sessions from each treatment (I-IV), before and after stimulation. The images were taken 2 hours before and 2 hours after the stimuli. I - Distilled water (A), II - nutrient solution (B), III - PEG solution (C), and IV - NaCl solution (D).

**Figure 3.**
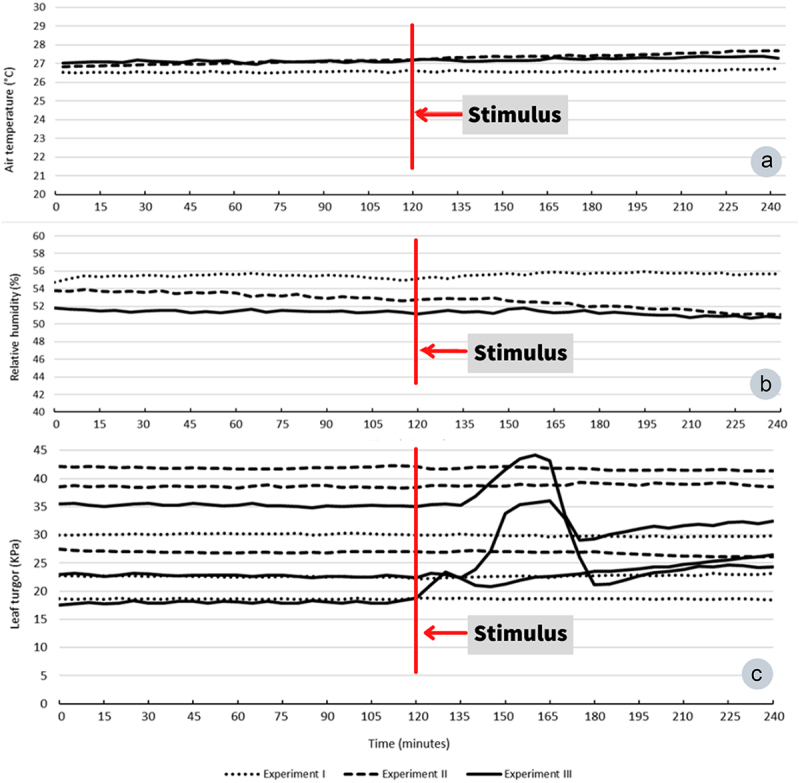
Average air temperature (A), relative humidity (B), and leaf turgor variation (C) recorded during all experimental sessions from each experiment. Dot-line (experiment I), dashed-line (experiment II), continuous line (experiment III). The red line indicates the moment of stimulation of plants. Experiment I: water; experiment II: nutritive solution; experiment III: PEG. In experiment IV the leaf turgor was not monitored.

As expected, plants stimulated with high water potential (Figures 2a & 2b) had no apparent effect on water status compared with the low water potential experiments (Figures 2c & 2d). On the other hand, the effects between PEG and NaCl on plants were distinct and clearly different each other after 2 hours of stimulation (Figures 2c & 2d). In this case, it was noticeable that PEG provoked a higher dehydration to plants than NaCl solution, despite the same water potential level. The leaf turgor of plants stimulated by high water potential had no alteration compared with low water potential stimulus with PEG (Figure 3c).

The air temperature (T) and relative humidity (RH) tended to be stable along the 4 hours duration of experimental sessions, showing no remarkable differences before and after the stimulation (Figure 3a, b), which did not affect the leaf turgor measured at the same time (Figure 3c).

Plants stimulated with high water potential (experiments I and II) had no significant leaf temperature alterations before and after both stimuli, despite a weak tendency for temperatures to slightly decrease after water application and slightly increase after the nutrient solution (Table 2). On the other hand, plants stimulated by low water potential had a significant leaf temperature increase after stimuli (Table 2).

**Table 2.** Leaf temperature and electrophysiological TS analysis from four experiments.

	I		II		III		IV	
	Before	After	Before	After	Before	After	Before	After
LT^1^	26.2133 ± 1.0634	25.8833 ± 1.6108	26.4238 ± 0.6252	26.7762 ± 1.9680	27.0000	29.7000**	24.5351 ± 1.6405	27.0676* ± 2.4143
p-value	=.319		=.477		≤.001		≤.001	
Δmv^2^	0.0015	0.0016	0.0004	0.0004	0.0022	0.0023	0.0010	0.0009
p-value	=.544		=.017		=0.877		=0.829	
Skew^3^	2.1200 ± 15.2724	−0.8853 ± 15.4015	0.0400	0.2300	−1.5661 ± 21.0430	7.2444 ± 47.2433	6.4557 ± 30.8791	4.0632 ± 25.3327
p-value	=.431		=.945		=0.203		=0.713	
Kurt^4^	397.3250	101.2900	247.1400	143.5900	155.4715	3695.61**	2091.2346 ± 2395.5050	2316.1251 ± 3207.9741
p-value	=.077		=.532		≤.001		=0.731	
PDF^5^	5.6573 ± 2.0147	5.6689 ± 1.7393	5.6463 ± 1.7788	5.5468 ± 1.6994	5.9022* ± 1.9045	3.2764 ± 1.2110	4.8281* ± 1.2300	3.8806 ± 1.0513
p-value	=0.978		=.804		≤.001		≤.001	
AC^6^	37.3950	44.1100	18.9200	16.0800	46.8096* ± 21.2402	30.6709 ± 19.7164	28.7300	38.1700**
p-value	=.080		=.175		≤.001		≤.001	
PSD^7^	1.7567 ± 0.2674	1.7533 ± 0.3267	1.7476 ± 0.2994	1.7619 ± 0.4566	1.6500 ± 0.3602	2.1254* ± 0.3271	1.900 ± 0.3914	1.9410 ± 0.4235
p-value	=.957		=.846		≤0,001		=0.560	
ApEn^8^	0.7783	0.5996	0.8073 ± 0.3136	0.8876 ± 0.4169	0.8594* ± 0.3874	0.1475 ± 0.2099	0.6270	0.3560
p-value	=.651		=.329		≤.001		=.052	

Means followed by * differ significantly according to paired t-test (*p* ≤ .05). Values represent the mean ± SD. The Wilcoxon Signed Rank Test (*p* ≤ .05) was used for data that did not show a normal distribution. Medians followed by ** differ significantly. The values represent the median. ^1^Leaf temperature in ºC (*n* = 30). ^2^Voltage variation (*n* = 30). ^3^Skewness (*n* = 30). ^4^Kurtosis (*n* = 30). ^5^PDF (*n* = 26). ^6^Autocorrelation (*n* = 30). ^7^Power Spectral Density’s β exponent (*n* = 30). ^8^Approximate entropy (*n* = 30). Experiment I (water); II (nutrient solution); III (PEG); and IV (NaCl).

By statistically comparing before and after each stimulus (Table 2), as expected, it was not possible to identify any significant difference (*p* ≥ .05) for any parameter in the water treatment. The same was observed for the treatment with nutrient solution. Regarding treatments with osmotic stimuli, leaf temperature was significantly higher after NaCl and PEG application. In the treatment with NaCl, the PDF exponent was significantly higher before the stimulus, and the autocorrelation was higher after PEG application. For PEG treatment, kurtosis and PSD exponent values were significantly higher after stimulation, while PDF exponent, autocorrelation, and ApEn were higher before stimulation.

**Figure 4.**
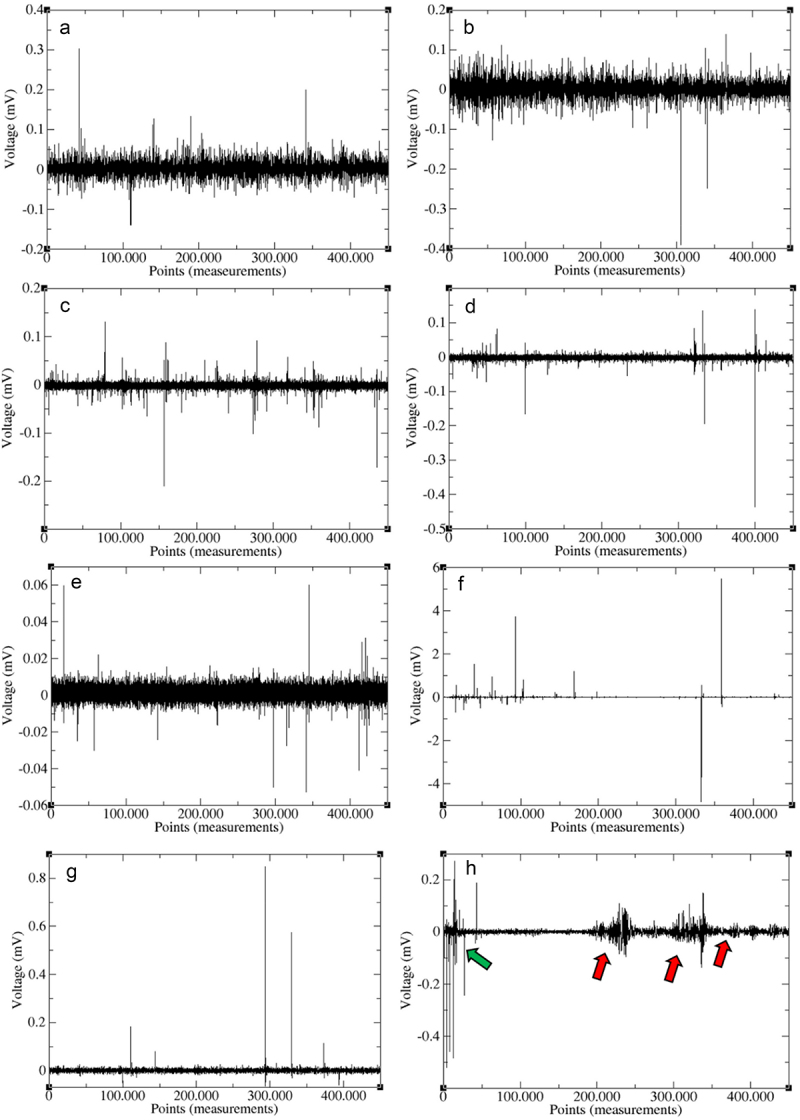
Electrophytograms before and after each of the four stimuli given in experiments I, II, III and IV, showing the voltage variation (mV) of plant electrome during 2 hours of each data collecting (2h = 450.000 points of the TS using sample rate of 62,5 hz). Electrophytograms before (A) and after (B) water, before (C) and after (D) nutrient solution, before (E) and after (F) PEG, and before (G) and after (H) NaCl solution.

The visual analysis of voltage oscillation (ΔV) in the time domain of all the electrophytograms shows that the electrome of each plant is unique (as one can note in few examples in Figure 4a, c, e, g). Voltage oscillations amplitudes tended to remain the same in each electrophytogram, with the appearance of a few peaks of amplitude a little greater than the more frequent oscillations, which occur both before and after the stimulus.

Generally, the electrome before any stimuli are very constant, with major voltage oscillations occurring between ±0.05 mV and with some few peaks (spikes) with higher amplitudes that usually do not pass from ±0.5 mV, despite few exceptions (Figure 4a, c, e, g). However, after the stimulation, there are an increase in frequency and in size of peaks of higher amplitudes (spikes), especially after low water potential solutions (PEG and NaCl) (Figure 4f, h). In these cases, there are some peaks with amplitudes that passes the ±5 mV (Figure 4f), sometimes reaching 10 mV in some electrophytograms (data not shown). After NaCl stimulation, appeared two very distinguishable patterns of voltage oscillation in almost all plants, that is the intense electrome activity in first 20–30 minutes right after stimulation (Figure 4h, indicated by green arrow) and the waves with long period (long time duration) with many peaks with high amplitude combined (inserted) in these long waves (Figure 4h, indicated by red arrows). Moreover, when the peaks (both before/after any stimuli) are amplified, it is possible to see that they are waves, usually very similar to action potentials (APs) and variation potentials (VPs).

**Figure 5.**
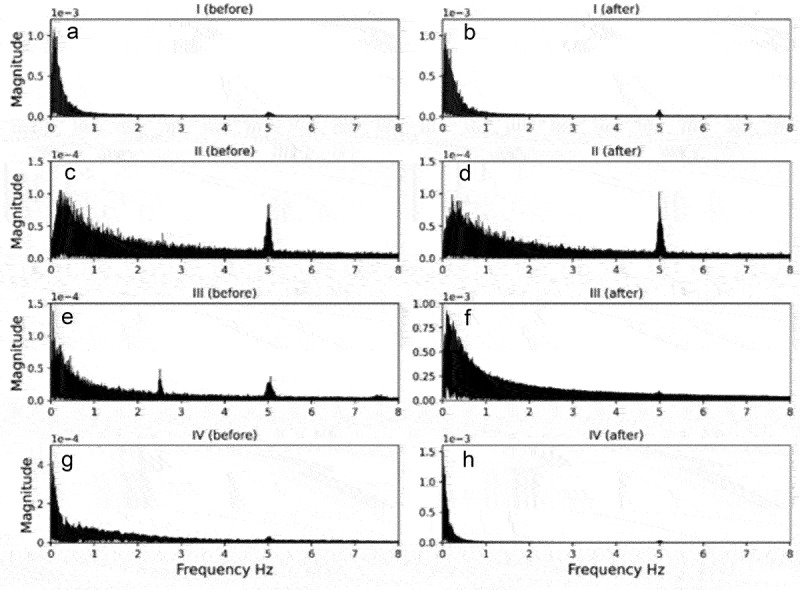
Example of plant electrome FFT before (A) and after (B) water, before (C) and after (D) nutrient solution, before (E) and after (F) PEG, before (G) and after (H) NaCl.

The FFTs of electromes before and after are shown in Figure 5, where is possible to see that the majority of voltage variations frequencies are from 0 to 3 Hz, specially concentrated between 0 to 1 Hz. The peaks in 5 Hz (and 2,5 Hz) that is clear in many FFTs graphs are consequence of noises from environment and from MP36 device. These frequencies are very clear in reference electrode FFTs, that was not inserted in any plant (data not shown). Thus, the frequencies of 5 Hz and 2.5 Hz, and also 7.5 Hz (present in some electromes FFTs that is not present in these examples of FFT graphs) are not from plant electrical activity.

The FFTs of electromes before any stimuli reinforces that each plant electrome is unique, with specific traits, in this case specific frequencies composition (Figure 5a, c, e, g). It is also noticeable that the maximum magnitude of frequencies of the electromes before any stimulation were the same (10^−4^). The comparison between FFT of electrome before and after stimulation with water and nutrient solution have no apparent differences, despite the differences among the FFTs shapes of the treatments. On the other hand, it is possible to see that after PEG and NaCl the frequencies distribution tended to be more concentrated between 0 and 1 Hz, with magnitude of frequencies being increased from 10^−4^ to 10^−3^ (Figure 5f, h). This pattern of FFT results also occurred in the majority of plants from each treatment, before and after the application of the stimuli.

**Figure 6.**
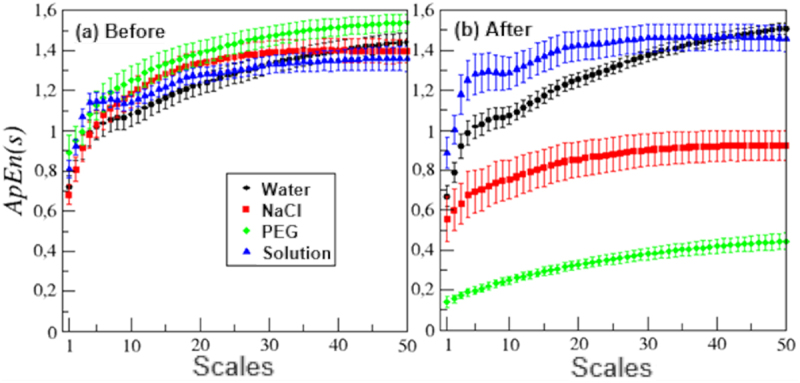
Multiscale approximate entropy mean (ApEn (S)) before (a) and after (b) four different stimuli.

The multiscale approximate entropy (ApEn(s)) analysis shows that electrome of plants before any stimulation was quite similar in values and curve shapes, since the most of ApEn(s) values in most of the scales are sharing the same space, with error bars overlapping in most of cases (Figure 6a). However, it is evident a small difference in shapes of ApEn(s) in the first 10 scales before water and nutrient solution (Figure 6a) when compared to ApEn(s) of plants before PEG and NaCl (Figure 6a). Such shape also remains in ApEn(s) curves after stimulating plants with water and nutrient (Figure 6b).

Comparing the ApEn(s) before and after water treatment, there were any statistical differences of values in all 50 scales (Figure 6a, b). The unique difference in ApEn(s) before and after water was a little increase/decrease in error bars sizes. The ApEn(s) values were statistically different between scales 7 and 36 comparing electrome of plants before and after nutrient solution stimulation and the error bars increased in all the 50 scales (Figure 6a, b).

The ApEn(s) after PEG stimulation had all values drastically decreased compared with ApEn(s) measured before, with values ranging from 0.8–1.6 (before) to 0.2–0.4 (after) (Figure 6a, b). Another tendence after PEG was the error bar size reduction in al the 50 scales (Figure 6a, b). The NaCl solution also led to a significant decrease in ApEn(s) values across nearly all scales, except for the first scale (Figure 6a, b). The error bars of ApEn(s) values increased in all the 50 scales after stimulation with NaCl (Figure 6a, b).

**Figure 7.**
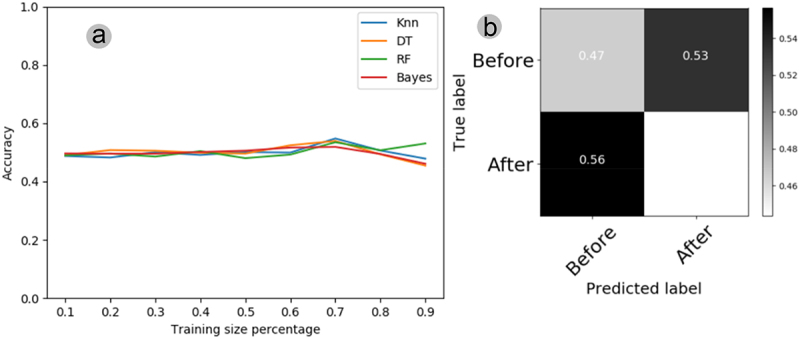
(A) learning curves depict the plant electrome before and after water treatment, employing four algorithms (KNN, DT, RF, Bayes) and varying training set sizes from 10% to 90% of the original time series previously processed through AI. (B) Confusion matrix for the electromes before and after water treatment, utilizing the KNN algorithm with 70% of the training set from the original time series processed through AI. In cases where the classifier could not distinguish anything, each cell in the matrix should register 0.5.

The automatic classification of the electrome shows that the pattern recognition accuracy of all classifiers (KNN, DT, RF, and Bayes) oscillated around 50%, with training sets varying between 10% and 90% (Figure 7a). The confusion matrix illustrates the comparison between the electrophysiological data before and after the stimulus, generated by utilizing 70% of the original series to train the KNN algorithm. This algorithm demonstrated superior performance with a smaller training set, yielding the best results. In the matrix, it is possible to see that KNN recognized the electromes “before” the stimulus with 47% accuracy and confused 53% of the electromes “after” with those “before” (Figure 7b). On the other hand, this same classifier (KNN) recognized the electromes “after” the stimulus with 44% accuracy and confused 56% of the electromes “after” with those “before” (Figure 7). Thus, the confusion matrix shows that the best classifier failed to find stimulus-dependent patterns in the plant electrome.

**Figure 8.**
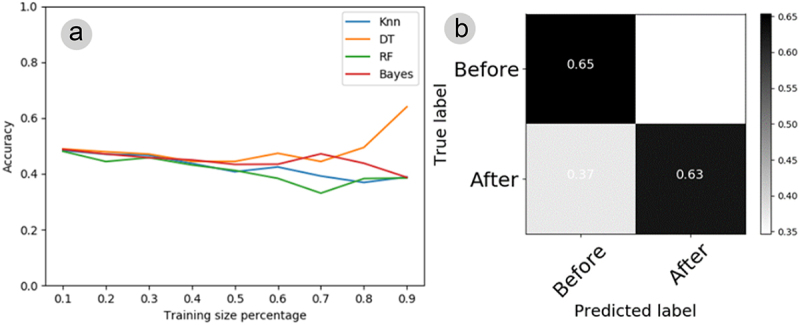
(A) learning curve depicting the plant electrome before and after nutrient solution application, employing four algorithms (KNN, DT, RF, Bayes) and varying training set sizes from 10% to 90% of the original time series previously processed through AI. (B) Confusion matrix for the electromes before and after nutrient solution treatment, utilizing the DT algorithm with 90% of the training set derived from the original time series previously processed through AI. In instances where the classifier could not discern anything, each cell in the matrix should indicate 0.5.

Figure 8a shows the electrome automatic classification of plants of experiment II (nutrient solution), employing four different algorithms (KNN, DT, RF, and Bayes) using training sets ranging from 10 to 90% of the length of the entire time series. The accuracy of pattern recognition in electrome oscillated around 50% (or below) in the training sets, excepting the DT, which reached a higher accuracy of 65% pattern recognition using a 90% training set. The confusion matrix between the data before and after the stimulus shows that the DT was able to correctly recognize 65% of the electromes before but confused 35% of the electromes “after” with those “before” (Figure 8b). On the other hand, the same algorithm recognized the electromes after the stimulus with 37% accuracy but confused 63% of the electromes “after” with those “before” (Figure 8b). Therefore, despite recognizing with up to 65% accuracy, this result shows that the DT classifier confused a considerable percentage of the electromes before and after the stimulus.

**Figure 9.**
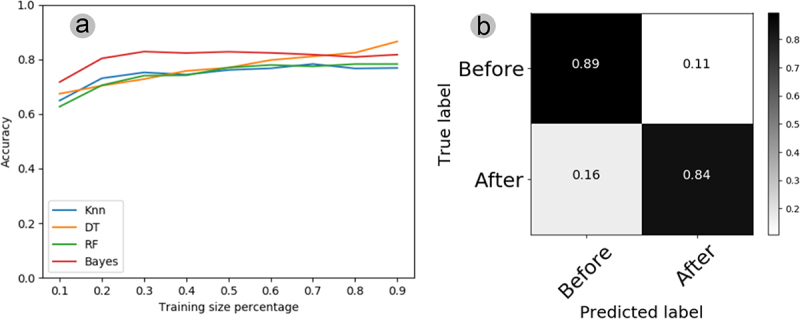
(A) learning curve illustrating the plant electrome before and after PEG exposure, employing four algorithms (KNN, DT, RF, Bayes) and training sets varying from 10% to 90% of the original time series previously processed through AI. (B) Confusion matrix for the electrome before and after PEG treatment, utilizing the DT algorithm with 90% of the training set obtained from the original time series previously processed through AI. In cases where the classifier couldn’t discern anything, each cell in the matrix should register 0.5.

The classification employing KNN, DT, RF, and Bayes showed that the pattern recognition accuracy was considerable for all algorithms at all training size percentages in experiment III (Figure 9a). The DT and Bayes classifiers were the best algorithms to recognize patterns in the electrome of the plants after the stimulus, reaching more than 80% recognition accuracy even using small training sets, mainly in the case of the Bayesian classifier. The confusion matrix between the electromes from before and after the stimulus using 90% of the original series for training the DT algorithm (Figure 9b). This algorithm was the one that achieved the best results with a smaller training set. In the matrix, it is possible to observe that DT classifier recognized the electromes “before” (89% of accuracy) but confused 11% of the electromes “before” with “after”. On the other hand, the DT classifier recognized 84% of the electromes “after” but confused 16% of the electromes “after” with the “before” (Figure 9b). Thus, using these algorithms, it is possible to recognize patterns or differences between plant electromes before and after PEG osmotic solution (−2 MPa).

**Figure 10.**
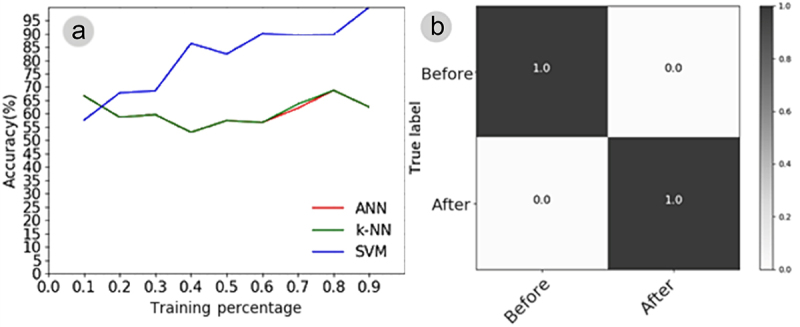
(A) learning curve illustrating the plant electrome before and after NaCl stimulation, employing three algorithms (ANN, KNN, SVM) and training sets varying from 10% to 90% of the original time series previously processed through IA. (B) Confusion matrix for the electrome before and after NaCl treatment, utilizing the SVM algorithm with 90% of the training set derived from the original time series previously processed through IA. If the classifier couldn’t discern anything, each cell in the matrix should show 0.5.

The automatic classification of the electrome employing three ANN, KNN, and SVM algorithms was adopted in experiment IV. The best algorithm was the SVM, which achieved up to 100% accuracy in recognizing the electrome pattern after stimulation, using a training set representing 90% of the original series (Figure 10a). The SVM confusion matrix showed that this classifier differentiated with 100% accuracy the differences in the electrome both before and after stimulation, with 0% confusion between the electromes “before” and “after” (Figure 10b). These results show that it is possible to recognize patterns or differences between plant electromes before and after saline application with very high accuracy using SVM, differently from ANN and KNN.

**Figure 11.**
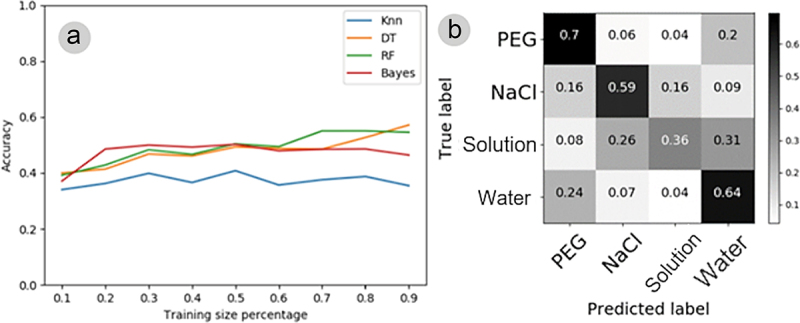
(A) learning curves depicting electrome patterns from four experiments (water, nutrient solution, PEG, and NaCl) employing four distinct classification algorithms (KNN, DT, RF, and Bayes) with training sets ranging from 10% to 90% of the original time series previously processed through IA. (B) Confusion matrix for the electromes collected from plants after undergoing stimuli from the four experiments, utilizing the DT algorithm with 90% of the training set derived from the original time series previously processed through IA. If the classifier couldn’t discern anything, each cell in the matrix should display 0.25.

The automatic classification of electromes of plants after the all the four stimuli was performed employing KNN, DT, RF, and Bayes, and training sets ranging from 10% to 90% of the original series. The learning curves of the four classifiers show different performances, with KNN presenting the lowest result, given the low accuracy and the tendency not to increase performance with more extensive training sets (Figure 11a). The other classifiers tended to improve the performance in recognizing electrome patterns after different stimuli. In this case, the DT classifier was the one that achieved the best result (59% accuracy) using a training set with 90% of the data (Figure 11a). Considering the performance of the DT classifier, a confusion matrix of the mixed electromes of the four experiments after stimulation was generated. It is possible to see that this classifier can differentiate the electromes of plants submitted to distinct stimuli with great accuracy (Figure 11b). DT managed to differentiate PEG stimulus better (70%), followed by water (4%), NaCl (59%), and nutrient solution (36%).

## 4. Discussion

As anticipated, non-harmful stimuli did not induce significant changes in either the plant’s water status or electrome dynamics, potentially indicating the habitual nature of such stimuli and their association with non-damaging effects.^38,46–48^ Nonetheless, it was crucial to confirm this observation, considering the electrome’s high sensitivity to even subtle stimuli and variations attributable to individual plant characteristics.^30,49^

Still, supporting the notion of high electrome responsiveness, detectable changes in multiscale ApEn measurements enabled automatic classification methods to identify alterations in the electrome following nutrient solution application (Figure 8). This evidence highlights that even commonplace and routine non-harmful stimuli can elicit changes in the bioelectrical activity of plants, manifesting in their electromes. It demonstrates the level of sensitivity exhibited by the electrical dimension of plants under non-stressful conditions, emphasizing the effectiveness of our classification methods in detecting subtle alterations in electrome activity.^40,50^

Likely, the increase in nutrient concentration within the root medium served as a more substantial stimulus signaling to the shoot than solely distilled water.^11,51^ Moreover, the absorption and translocation of nutrients are closely associated with alterations in cell membrane potential, given that nutrient salts dissolved in water possess electrical charges, and their entry into cells relies on ion exchanges that induce changes in membrane potential.^29^ The movement of water and nutrients through the vascular tissues of plants, along with alterations in turgor pressure, is recognized to induce changes in electrical activity. These processes are inherently interconnected and inseparable.^5,6,8,29^ However, the salts present in the solution decrease the osmotic potential of the root medium, which might have served as a mild osmotic stimulus. Nevertheless, this alteration could be substantial enough to modify electrophysiological processes and long-distance signaling within the plant.^6–8,31^

On the other hand, On the contrary, the detrimental stimuli induced by PEG and NaCl solutions, both at −2 MPa (distinct stimuli with the same water potential), notably altered the plant’s water status characteristics. The effect was more pronounced 2 hours after exposure to PEG (Figure 2c) compared to the observation 2 hours after NaCl exposure (Figure 2d). The leaf turgor variation also exhibited significant changes after the PEG stimulus in contrast to those observed with distilled water or nutrient solution (Figure 3c), indicating a loss of turgor regulation.^52,53^ The dehydration state of the plants suggests that PEG might have caused not only a reduction in stomatal opening and transpiration but also a withdrawal of water from the root tissues. PEG 6000, being a large polymer, does not penetrate the cell wall or plasmalemma but possesses a high affinity for water, thereby reducing the water potential. Consequently, water could have moved from both the apoplast and symplast of the roots to bind with PEG molecules present in the external environment due to differences in water potential between plant roots and the external environment, leading to intense dehydration (Figure 2c).^47,53,54^

The differences in the characteristics of the beans’ electrome after applying the PEG solution are distinctly visible in the electrophytograms. This includes the emergence of high-amplitude peaks in voltage variation, often hundreds or even thousands of times higher (Figure 4e) than the normal electrome activity observed prior to the PEG stimulus (Figure 4f). This significant alteration in the electromes post-PEG stimulus likely impacted all the parameters used to characterize the electrome, as described below. The noticeable changes in Fast Fourier Transforms (FFTs) also indicate a shift in frequency composition after PEG application, leading to a prevalence of lower frequencies (ranging from 0 to 1 Hz) with higher power (ranging from 10^−4^ to 10^−3^). It is worth noting that upon zooming in on these peaks, they exhibit wave-like shapes reminiscent of well-known action potentials and variation potentials.^6,8^

However, the mean of voltage variation was unaffected by PEG. Skewness displayed a tendency to increase (from −1.56 to 7.24) after PEG, yet this difference lacked statistical significance, possibly due to high standard deviations (SDs). Conversely, kurtosis measurements indicate a distinct distribution of voltage variation events following the stimulus, revealing a notable trend toward increased kurtosis values (from 155.4 to 3695.6) (Table 2). This trend signifies a discernible pattern in the alteration of the distribution of voltage variation events subsequent to the PEG stimulus. In this scenario, the histograms demonstrated greater leptokurtosis, indicating a more extended tail than that of a normal curve. This suggests the occurrence of infrequent high-amplitude events as opposed to more frequent events of lower amplitude.

The power law function ((∆*V*)~|∆*V*|^−µ^) emerged as the most fitting function to accurately describe the distribution of voltage variation in plant electromes across the four experiments, both before and after stimulation. This phenomenon cannot be effectively described by Gaussian (normal distribution), logarithmic, exponential, trigonometric, or other functions. This is a crucial observation in itself, but its significance amplifies when considering the results derived from measuring the PDF exponent. The substantial decrease (from 5.9 to 3.2) in the mean values of the µ exponent, validated through paired t-tests post-PEG application, indicates a tangible impact of PEG on the distribution of voltage variations within the electrome. This alteration suggests a trend toward a smoother slope (less steep) in the fitted line. Consequently, after PEG exposure, the probability density function tends to display a more extensive and thicker tail, depicting a less concentrated distribution around the mean and mode. This phenomenon suggests the occurrence of more infrequent events following the stimulus.^33,34,55–57^

The tendency of the means of the PDF exponent to hover around 3, particularly the PDF exponent values observed in 15 plants (ranging between 1 and 3), indicates the signatures of highly complex scale-free systems. These systems exhibit a high level of information distributed across all scales and are associated with self-organized criticality.^33,34,55–58^ Previously, through visual analysis of the electrophytograms, there was a hypothesis that the exceptionally high peaks in the electromes following PEG exposure might possess such characteristics. The appearance of various peak sizes implied a pattern where “the larger the peak, the less frequent it is,” which aligns with the typical behavior of a scale-free phenomenon, exhibiting scale invariance or a Wiener process.

A diverse array of phenomena can be effectively described by power laws. These include phase changes in the physical state of matter, the formation of dunes, coastal landscapes, raindrops, rice piles, avalanches, earthquakes, volcanic activity, meteorological influences, species evolution, population dynamics, and numerous other occurrences. These phenomena rely on various elements and forces that interact with each other, giving rise to critically self-organized patterns that transcend scale. These patterns tend to replicate themselves across different scales, often spanning from microscopic to macroscopic levels, without external agent control. They rely on transition points and discontinuities in the functioning and organization of such complex systems. These characteristics are commonly associated with the accumulation and dissipation of mass and/or energy, or with the plasticity and resilience of systems.^56,59–63^

Power law distributions are also associated with the effectiveness of information processing, transmission, and storage, a concept observed in the organization and function of the nervous systems of animals, including humans. In this context, both the distribution of neuronal connections (synapses) and the functioning of the bioelectrical activity in the central nervous system (CNS) adhere to a power law, contingent on the situation.^56,64,65^ The distribution of the brain’s electrical activity following a power law appears linked to alterations and transitions in the dynamics of functioning, typically occurring during cognitive processes in healthy individuals, such as learning, motor coordination, and memory. These characteristics were likely selected in evolutionary terms because they optimize the efficacy of information transmission, processing, and storage across all scales, from neurons to the entire CNS. This theory is known as the “neural criticality hypothesis”.^56,62,66–68^

Moreover, there is more evidence indicating that the bioelectric activity of plants and algae follows a power law, contingent upon the stimulus or environmental conditions.^33,34,69^ In the case of plant electrome, the power law distribution and self-organized criticality (SOC) behavior are explained as emergent phenomena related to peaks with amplitudes inversely proportional to their frequency. This phenomenon is likely associated with alterations in information transmission and processing, variations in physiological states, and energy distribution. We hypothesize that harmful stimuli (such as PEG/NaCl) provoke avalanches of ionic species, particularly Ca^2+^ waves, accompanied by reactive oxygen species (ROS) waves, changes in vessel pressure, alterations in cell turgor pressure, and synchronous triggering of membrane potential.^6,8,14,19,30,51,70–72^

A significant increase in the mean values of PSD exponent was observed, rising from 1.65 to 2.12 after exposure to PEG (Table 2). This shift indicates a tendency toward a “darker” noise color following the stimulation, suggesting that the system (electrome) increased random frequencies while reducing organization. Prior to PEG exposure, the electrome’s PSD exhibited a more organized frequency distribution across the spectrum. Post-stimulation, however, there appeared to be a reduction in the number of frequencies, as seen in the FFTs (Figure 4e, f), which displayed a restriction of lower frequencies with higher power. Moreover, considering that PSD is inversely proportional to frequency (PSD = 1/fβ), its relationship with FFTs is direct. Therefore, as β = 1 represents pink noise and β = 2 denotes brown noise, the trend toward “darker” noise was observed due to a shift in the PSD exponent. Pink noise is indicative of highly complex systems characterized by self-organization, SOC behavior, scale invariance, and persistence, aligning with the previous findings from experiment III.^33,34,55,56,67^

Autocorrelation values exhibited a significant decrease following the PEG stimulus, from 46.8 to 30.6 (Table 2). This reduction suggests that events within the electrome were inclined to influence subsequent events over a shorter duration in the future. In other words, the voltage variation in the electrome tended to display more short-duration events. This change also implies a shift in the information content within the electrome after the PEG stimulation. This observation aligns with alterations seen in both the PSD exponent and FFT, given that the PSD is associated with the autocorrelation function, as described by the Wiener-Khinchin theorem.^55,56,67^

However, the ApEn analysis supports the notion that alterations in the organization and complexity of electrical activity take place following the stimulation. It was observed that the mean ApEn values shifted from 0.85 (pre-stimulation) to 0.14 after the PEG treatment, and this difference was statistically significant according to the paired t-test (*p* ≤ .05). The multiscale entropy measurements (ApEn(s)) further validate this outcome. It is clear that the application of PEG induced changes in the complexity and organization of the bean electrome, as evidenced by a decrease in complexity across all 50 measured scales (Figure 6a, b).^73,74^

The reduction in complexity suggests that the stimulus induces changes in the composition of voltage variation events, leading to increased organization, decreased heterogeneity, and a lower level of randomness within the electrome. Therefore, although the complexity (as measured by ApEn and ApEn(s)) has decreased, this does not necessarily imply that the electrome has become less complex in terms of the richness of patterns. On the contrary, this reduction in ApEn values indicates that the electrome starts displaying a richness of identifiable complex patterns and less random noise compared to correlated noises.^73–77^

The decrease in complexity must also be a consequence of the emergence of high amplitude peaks in the electrome, which promotes a greater level of organization in the system by reducing randomness, thus adding more regular patterns to the electrome dynamics. Saraiva et al.^34^ also found a decrease in the electrome complexity of soybean plants after an osmotic stimulus. The authors considered that the lower ApEn values could be both an indication of electrome dynamic stability decrement, and an indication of the deterioration of the physiological processes caused by osmotic stress.

Regarding the signal classification potential, the decision tree (DT) algorithm emerged as the most effective classifier in experiment III, displaying superior performance indicated by its ascending curve and high accuracy in pattern recognition among the four classifiers tested (Figure 9a). The confusion matrix underscores the DT classifier’s robust ability to discern plant electromes before and after PEG stimulation, achieving an 89% accuracy in recognizing electromes before and 84% after PEG (Figure 9b). This proficient discrimination between pre- and post-PEG electromes highlights the substantial impact of PEG stimulus on bean electromes, aligning with the earlier discussed results. Furthermore, the level of accuracy in pattern recognition is consistent with findings from prior studies on automatic classification of plant bioelectrical activity.^40,78–80^ This underscores the potential use of plant electromes in early stress detection when combined with automatic classification algorithms.

The effects induced by the NaCl solution on plant electrome and plant water status differed from those caused by PEG, although exhibiting certain similarities. Notably, despite both solutions sharing the same water potential (−2 MPa), NaCl induced less dehydration in plants (Figure 2D) compared to PEG solution (Figure 2c), indicating distinct stress conditions despite the similarity in water potential intensity. Before NaCl stimulation, the leaf temperature was 24.5°C, which increased to 27°C after exposure to NaCl, suggesting that saline stress significantly hampered heat dissipation through leaf transpiration.^52^ This increase in temperature was statistically significant based on a paired t-test (*p* ≤ .05) and mirrored the leaf temperature elevation observed following the PEG stimulus.

By visual analysis of electrophytograms, it was evident that NaCl had an impact on the plant’s electrome, notably by increasing the number of high-amplitude peaks (Figure 4g, h). However, the most intriguing alterations observed in the electrome after NaCl treatment included a sudden surge of peaks within the initial 20–30 minutes following NaCl application (Figure 4h, green arrow), along with the emergence of another pattern characterized by multiple closely spaced peaks, forming prolonged wave-like structures (Figure 4h, red arrows). These two distinct patterns were uniquely observed in the electrome of plants exposed to NaCl among all the experimental conditions tested in our four experiments.

These unique and particularly intriguing patterns manifest as a consequence of intense electrical activity characterized by successive waves with high voltage amplitudes, closely resembling action potentials and variation potentials. This observation is consistent with existing knowledge that salts and alterations in the osmotic potential of the root medium stimulate the generation of electrical signals for long-distance communication, transmitting through the plant’s vascular tissues, thereby passing through the stem.^6,8,9,11,51^

The alteration in the electrome subsequent to NaCl exposure resulted in changes in the Fast Fourier Transforms (FFTs), leading to a reduction in frequency composition and an amplification in the power of the frequencies (Figure 5g, h). This phenomenon bears resemblance to the alterations observed in the FFTs of the PEG experiment (Figure 5e, f). As previously discussed regarding PEG, these FFT alterations after NaCl are likely a consequence of the increase in both the frequency and magnitude of peaks. These findings align with other analyzed parameters such as the PDF µ exponent and autocorrelation, along with the observed decreasing trend in both ApEn and ApEn(s) values (Fig A,B).

Interestingly, despite the observed alterations in various parameters after NaCl exposure, the PSD exponent did not display any significant changes as anticipated from the aforementioned results. Notably, the β exponent values around 1.9 for both before and after the salt stimulus indicate that the electrome exhibits characteristics akin to brown noise, suggesting a system with less complexity than pink noise.^73,74^ Conversely, the µ exponent values near 3 following NaCl exposure (Table 2) suggest an increase in the electrome’s complexity. This places the electrome within the range of 1 < µ < 3, indicative of highly complex systems associated with self-organized criticality (SOC) behavior, scale invariance, information processing/transduction, phase transition, alterations in physiological status, among numerous other implications, as discussed later in the context of the results obtained from the PEG experiment.^73,74^

The trend toward decreased ApEn after exposure to NaCl was evident, although it did not reach statistical significance with the employed tests (Table 2). However, the analysis of ApEn(s) revealed a drastic and significant reduction in complexity across 49 scales, highlighting a clear decline in the electrome’s complexity. This emphasizes that ApEn(s) might be a more suitable measure for assessing plant electrome complexity, similar to its efficacy in EEG and ECG analyses.^73^ The observed decrease in complexity, as measured by ApEn, suggests a reduction in irregularities and an increase in identifiable patterns within the electrome, signifying a more organized and potentially complex phenomenon, depending on the interpretation applied.^34,73–76^ Conversely, reductions in ApEn(s) are typically associated with diseases and physiological deterioration in systems, including plant physiology. This aligns with the stress induced by the intense osmotic/saline solution (NaCl at −2 MPa), which likely disrupted the physiological processes of the plants, evident in the photographic evidence (Figure 2d).^34,73–76^ Other considerations made in the discussion concerning the effects of PEG on the electrome are also relevant to the observed decrease in complexity levels measured by ApEn/ApEn(s).

The automatic classification of the electrome yielded even more remarkable results (Figure 10) compared to the classification based on PEG electrophytograms (Figure 9). Among the algorithms, the Support Vector Machine (SVM) exhibited the ability to differentiate the electrome of plants after stimulation by the saline solution with 100% accuracy (Figure 10). This unequivocally demonstrates that the features of plant electrome were indeed altered by NaCl, as revealed by other parameters. Furthermore, it underscores the capability of automatic classification algorithms in effectively distinguishing between the electrome of plants subjected to different conditions.

Pereira et al.,^40^ employing similar methodologies for automatic electrome classification, demonstrated that SVM (Support Vector Machine) achieved the highest accuracies in detecting different stimuli (80% for low light and low osmotic potential, and 90% for low temperature). Following this, KNN (K-nearest neighbors) achieved 74% accuracy in low light detection, 81% in osmotic detection, and 90% accuracy in low-temperature detection. Other algorithms examined in the same study, such as Optimal Path Forest (OPF) and Convolutional Neural Network (CNN), also exhibited satisfactory detection accuracies in certain cases, while the Artificial Neural Network presented the least favorable results. Notably, both KNN and SVM algorithms were successful in detecting ‘all’ stimuli when presented in a mixed format.^40^

A recent study reported that the automatic classification of plant physiological status based on its electrical activity can be achieved in real-time outside a Faraday cage (in a greenhouse) with high accuracy levels. This study reached 94% accuracy in day/night detection and 98% accuracy in detecting water stress using the Gradient Boosted Tree (GBT) algorithm.^81^ Furthermore, this research emphasized that real-time monitoring of plant electrical activity dynamics can serve as an effective method for the early detection of water stress, particularly because changes in electrical activity may precede visible symptoms and alterations in leaf turgor measured by Yara water sensors.^81^

Further research has shown that distinct patterns of electrical activity, as measured by electrical resistance and variance of the olive tree stem, can serve as bioelectrical signatures representing varying intensities of water stress.^82^ This study demonstrated the feasibility of automatically classifying electrical activity, enabling the differentiation between intense and moderate water stress from control groups (no water stress). Specifically, the use of binary classifiers allowed the identification of the control group with 93% accuracy, while the mid-stress group achieved 76%, and the high-stress group reached 80% accuracy.^82^

Our results align with the findings of Tran et al.^81^ and Comparini et al..^82^ They indicate that variations in plant electrical activity in response to different levels and conditions of water availability or unavailability may serve as early indicators of plant water status compared to conventional devices currently used for monitoring, such as leaf turgor pressure probes (Yara water sensors). Importantly, these electrical activity alterations exhibit superior accuracy in distinguishing between conditions, even in scenarios where different stress types, like salt and osmotic stress with equivalent water potential, need differentiation.

Moreover, this study also demonstrates the potential of utilizing automatic classifiers on plant electrome to identify various stimuli administered to common beans, effectively distinguishing patterns even among plants exposed to different water potential stimuli (Figure 11). This strongly suggests that bean electrome presents stimulus-specific patterns, potentially useful for detecting various stressful conditions, as indicated in previous studies.^30,33,34,40,41,78–80,81,83,84^

Furthermore, the characterization of plant electrome through multiple time series analyses suggests that various parameters could serve to identify plant water stress. These findings provide new insights and perspectives into the features and dynamics of plant electrome under different conditions, offering potential practical applications for the early detection of such adverse conditions.

## 5. Conclusion

The electrophysiological responses in common beans display characteristics that are contingent upon the stimuli, even when exposed to stimuli of similar intensity. The complexity of the bean’s electrophysiological profile remains intricate across various scenarios, undergoing alterations based on the nature of the stimuli applied, resulting in a decrease in Approximate Entropy (ApEn) and ApEn(s) values during periods of stressful stimulation. Despite the inherent complexity of the bean’s electrophysiological reactions, our investigation demonstrates the ability to differentiate between the effects of distinct stimuli by employing automatic classification algorithms on electrophysiological time series data, even when the electrophysiological data from the four stimuli are combined. Thus, our initial hypothesis stands validated. The outcomes of our study strongly suggest the potential of utilizing electrophysiological data as an indicator for evaluating the physiological condition of plants, particularly concerning water availability. This potential indicates promising pathways for integrating our methodologies, encompassing the acquisition, analysis, and recognition of patterns within plant electrophysiology, thereby facilitating early detection of water, osmotic, and saline stresses.
